# Comparison of Direct and Video Laryngoscopes during Different Airway Scenarios Performed by Experienced Paramedics: A Randomized Cross-Over Manikin Study

**DOI:** 10.1155/2020/5382739

**Published:** 2020-02-18

**Authors:** Kurt Ruetzler, Lukasz Szarpak, Jacek Smereka, Marek Dabrowski, Szymon Bialka, Lauretta Mosteller, Agnieszka Szarpak, Kobi Ludwin, Marzena Wojewodzka-Zelezniakowicz, Jerzy Robert Ladny

**Affiliations:** ^1^Cleveland Clinic, Departments of General Anesthesiology and Outcomes Research, Anesthesiology Institute, Cleveland, OH, USA; ^2^Lazarski University, Medical Simulation Center, Swieradowska 43, Warsaw, Poland; ^3^Wroclaw Medical University, Department of Emergency Medical Service, Parkowa 34, Wroclaw, Poland; ^4^Poznań University of Medical Sciences, Chair and Department of Medical Education, Dabrowskiego 12, Poznan, Poland; ^5^Medical University of Silesia, Department of Anesthesiology, Intensive Care and Emergency Medicine, 3-go Maja 13-15, Zabrze, Poland; ^6^Cleveland Clinic, Department of Outcomes Research, Anesthesiology Institute, Cleveland, OH, USA; ^7^Polish Society of Disaster Medicine, Swieradowska 43, Warsaw, Poland; ^8^Medical University of Bialystok, Department of Emergency Medicine, Szpitalna 37, Bialystok, Poland

## Abstract

*Introduction*. Airway management plays an essential role in anaesthesia practice, during both elective and urgent surgery procedures and emergency medicine.

**Aim:**

The aim of the study was to compare Macintosh laryngoscope (MAC), McGrath, and TruView PCD in 5 separate airway management scenarios.

**Methods:**

This prospective cross-over simulation study involved 93 paramedics. All paramedics performed intubation using direct laryngoscope (MAC), McGrath, and TruView PCD video laryngoscopes. The study was performed in 5 different scenarios: (A) normal airway, (B) tongue oedema, (C) pharyngeal obstruction, (D) cervical collar stabilization with tongue oedema, and (E) cervical collar stabilization with pharyngeal obstruction.

**Results:**

In scenario A, the success rate was 99% with MAC, 100% with McGrath, and 94% with PCD. Intubation time was 17 s (IQR: 16–21) for MAC, 18 s (IQR: 16–21) for McGrath, and 27 s (IQR: 23–34) for PCD. In scenario B, the success rate was 61% with MAC, 97% with McGrath, and 97% with PCD (*p* < 0.001). Intubation time was 44 s (IQR: 24–46) for MAC, 22 s (IQR: 20–27) for McGrath, and 39 s (IQR: 30–57) for PCD. In scenario C, the success rate with MAC was 74%, 97% with McGrath, and 72% with PCD (*p* < 0.001). Intubation time was 44 s (IQR: 24–46) for MAC, 22 s (IQR: 20–27) for McGrath, and 39 s (IQR: 30–57) for PCD. In scenario C, the success rate with MAC was 74%, 97% with McGrath, and 72% with PCD (*p* < 0.001). Intubation time was 44 s (IQR: 24–46) for MAC, 22 s (IQR: 20–27) for McGrath, and 39 s (IQR: 30–57) for PCD. In scenario C, the success rate with MAC was 74%, 97% with McGrath, and 72% with PCD (*p* < 0.001). Intubation time was 44 s (IQR: 24–46) for MAC, 22 s (IQR: 20–27) for McGrath, and 39 s (IQR: 30–57) for PCD. In scenario C, the success rate with MAC was 74%, 97% with McGrath, and 72% with PCD (

**Conclusions:**

The McGrath video laryngoscope proved better than Truview PCD and direct intubation with Macintosh laryngoscope in terms of success rate, duration of first intubation attempt, number of intubation attempts, Cormack-Lehane grade, percentage of glottis opening (POGO score), number of optimization manoeuvres, severity of dental compression, and ease of use.

## 1. Introduction

Advanced airway management is crucial in the treatment of severely injured or sick patients [1]. Endotracheal intubation is considered the preferable technique to secure and protect an airway, especially in the out-of-hospital emergency setting. Endotracheal intubation might be challenging, especially if performed by relatively inexperienced providers and prolonged intubations lead to a higher risk of adverse respiratory events and increased risk of airway injuries [1–3]. Unrecognized esophageal intubation is associated with even worse clinical outcomes and may cause even death [1, 3, 4].

Endotracheal intubation in the out-of-hospital setting is especially challenging, as paramedics have to deal with difficult circumstances and suboptimal positions [1]. Direct laryngoscopy using a Macintosh blade (MAC) is the widely used standard technique for endotracheal intubation, but this technique requires a high level of training and personal skills [1, 2, 4, 5]. Video laryngoscopy was introduced into clinical practice to ease endotracheal intubation and might be especially useful in less experienced providers like paramedics. Video laryngoscopes are equipped with a camera on the tip of the blade, enabling better visualization of the airway anatomy and ultimately making it easier to visualize the entrance to the larynx [3, 6]. Several video laryngoscopes have recently been developed and are currently commercially available [3].

The McGrath video laryngoscope (Aircraft Medical, Edinburgh, United Kingdom) is a portable video laryngoscope with Macintosh-style blades for paediatric and adult patients. It is in clinical use for many years now and is widely used in both in-hospital and out-of-hospital settings [7, 8]. The Truview PCD (referred to in this paper as PCD) system consists of a set of optical blades for neonates, paediatric patients, and adults with a built-in cleaning system, handles, blades with a dedicated Truview PCD camera, and a monitor with photo and video recording capabilities [9–11]. The Truview PCD offers a unique blade that provides a wide angle optical view and, using a prismatic lens, enables visualization of the larynx entrance (without requiring the alignment of the oral, pharyngeal, and tracheal axes) and confirmation of correct introduction and position of the endotracheal tube [9–11].

The aim of the study was to compare MAC, McGrath, and PCD in 5 different simulated airway management scenarios. Our hypothesis was that the video laryngoscopes are superior in terms of intubation success rate compared to MAC if used by paramedics in a manikin setting.

## 2. Material and Methods

The study protocol was approved by the institutional review board of the Polish Society of Disaster Medicine (approval no. 21.09.2018.IRB). After obtaining written informed consent, 93 paramedics were enrolled in the study. The inclusion criteria were as follows: more than 5 years of professional work experience, previous experience with endotracheal intubation using MAC, and no previous experience with any type of video laryngoscopes.

### 2.1. Study Protocol

All paramedics participating in the study underwent a 45-minute lasting lecture covering basic aspects of airway management using direct laryngoscopy and video laryngoscopy. Afterwards, paramedics were allowed to familiarize themselves with the McGrath and TruView PCD laryngoscopes and were asked to perform at least one successful intubation with each device. All intubations were performed on a MegaCode Kelly advanced life support manikin (Laerdal Medical, Stavanger, Norway).

Paramedics were then randomly assigned to 1 out of 3 groups.

1. Direct laryngoscopy using a Macintosh blade size 3 (MAC)
2. McGrath video laryngoscope equipped with a size 3 Macintosh blade (McGrath)
3. TruView video laryngoscope.

**Figure 1:**
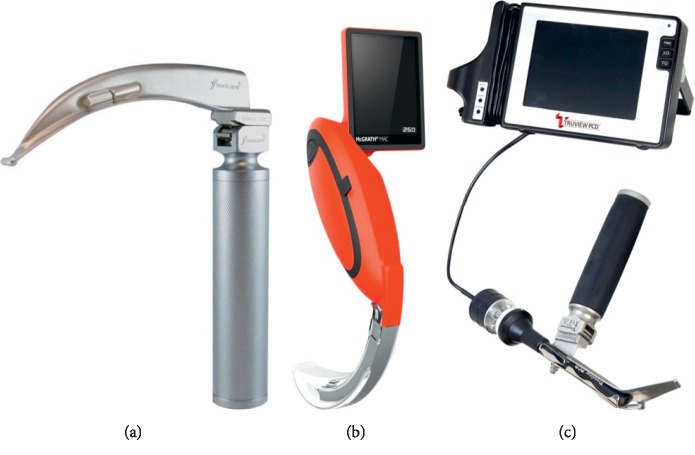
Laryngoscopes used in this study: (a) Macintosh laryngoscope; (b) McGrath MAC video laryngoscope; (c) TruView PCD video laryngoscope.

A 7 mm I.D. endotracheal tube (Heine USA Ltd., Dover, USA) (Figure 1) was used for all intubations.

Randomization was based on the Research Randomizer (http://www.randomizer.org) software. The airway of the manikin as well as the tubes was well lubricated. The tubes were equipped with a hockey-stick-shaped stylet, prepared by an experienced researcher. All study participants were allowed to adjust the stylet as desired. After randomization, paramedics were asked to perform 5 intubations in 5 subsequent airway scenarios.

1. Scenario A: normal airway.
2. Scenario B: tongue oedema.
3. Scenario C: pharyngeal obstruction.
4. Scenario D: cervical collar stabilization with tongue oedema. Collar stabilization was performed with a standard patriot cervical extraction collar (PatriotOessur Americas, Foothill Ranch, USA), applied to the manikin's neck by an independent instructor.
5. Scenario E: cervical collar stabilization with pharyngeal obstruction. Collar stabilization was performed with a standard patriot cervical extraction collar (PatriotOessur Americas, Foothill Ranch, USA), applied to the manikin's neck by an independent instructor.

Once the paramedic completed the initial 5 scenarios, the paramedic was asked to perform another 5 airway scenarios with an alternate technique in the same manner as described above. After the completion of all airway scenarios using the second airway technique, paramedics performed the final five airway scenarios with the third and remaining airway technique.

All scenarios were limited to a maximum of 1 intubation attempt and each intubation attempt was limited to a maximum of 60 seconds. To avoid any teaching bias, all paramedics performed the intubations alone and were not allowed to observe one another.

### 2.2. Measurements

The primary endpoint was the overall success rate. The secondary endpoints included duration of intubation attempt, Cormack-Lehane grade, POGO score, number of optimization manoeuvres, severity of dental compression, and ease of use.

The duration of an intubation attempt was defined as the time from grasping the airway device until the first successful ventilation of the lungs. Ease of use was assessed with a visual analogue scale score ranging within 1–100, with 1 indicating “extremely easy” and 100 indicating “extremely difficult.” The number of optimization manoeuvres was assessed and documented by observation by an independent researcher. POGO score and Cormack-Lehane classification were assessed by asking the paramedics after each intubation attempt [12].

### 2.3. Statistical Analysis

The sample size was based on expected differences of time to intubation and calculated with G × Power 3.1 using a two-tailed *t*-test (Cohen's *d* = 0.8, alpha error = 0.05, power = 0.95). With the minimum of 80 participants necessary, 93 paramedics were included to compensate for potential doubts.

The statistical analysis was performed with the Statistica software version 13.3EN for Windows (Tibco Inc., Tulsa, USA). The level of significance was set at the value of *p* < 0.05. Data are presented as number (percentage), mean ± standard deviation (SD), or median (interquartile range [IQR]), as appropriate. Nonparametric tests were used for the data that did not have normal distribution, which was tested with the Lilliefors test and the Shapiro–Wilk test. All statistical tests were two-sided. The one-way ANOVA on ranks was applied to compare the different times and to determine the statistical difference for each group (post hoc Bonferroni correction was used to counteract the problem of multiple comparisons).

## 3. Results

Ninety-three paramedics with a median of 8.5 years [IQR: 5.5–11] of experience participated in this study. All of the paramedics had a previous experience with direct laryngoscopy guided endotracheal intubation (median 54 intubations (IQR: 42–77)) and none had any experience with any video laryngoscope.

### 3.1. Scenario A: Normal Airway

Detailed results obtained in scenario A are presented in Table 1. The success rate of MAC, McGrath, and PCD was 99% versus 100% versus 94%, respectively (*p*=0.011).

The intubation time varied between 17 (IQR: 16–21) seconds for MAC, 18 (IQR: 16–21) seconds for McGrath, and 27 (IQR: 23–34) seconds for PCD. Cormack-Lehane score was best for McGrath and PCD and worst for MAC. A similar correlation was observed for POGO score, with a value of 81% during intubation with MAC and 100% with McGrath and PCD.

The ease of use averaged 20 (IQR: 11–23) for McGrath, 24 (IQR: 10–27) for MAC, and 31 (IQR: 17–35) for PCD.

### 3.2. Scenario B: Tongue Oedema

Results of scenario B are presented in Table 2. The intubation success rate was 61% for MAC, 97% for McGrath, and 97% for PCD.

The intubation time was 22 (IQR: 20–27) seconds for McGrath, 39 (IQR: 30–57) seconds for PCD, and 44 (IQR: 24–46) seconds for MAC.

Cormack-Lehane score was best for McGrath and PCD and worst for MAC. Average POGO score was the highest with 93% for McGrath, compared to 90% for PCD and 37% for MAC.

Endotracheal intubation with McGrath was associated with the fewest optimization manoeuvres in comparison with MAC (*p* < 0.001) and PCD (*p* < 0.001). McGrath also had the fewest number of tooth compressions in comparison with MAC (*p* < 0.001) and PCD (*p* < 0.001).

The ease of use averaged 74 (IQR: 50–80) for MAC, 34 (IQR: 26–47) for McGrath, and 46 (IQR: 38–61) for PCD.

### 3.3. Scenario C: Pharyngeal Obstruction

Results obtained in scenario C are presented in Table 3. The intubation success rate was 74% with MAC, 97% with McGrath, and 72% with PCD.

The intubation time with the studied methods varied between 21 (IQR: 19–29) seconds for MAC, 18 (IQR: 18–24.5) seconds for McGrath, and 30 (IQR: 23–39) seconds for PCD.

The best POGO score was obtained for McGrath and for PCD (100% each), and the worst for MAC (80%).

Endotracheal intubation with McGrath was associated with the fewest optimization manoeuvres in comparison with MAC (*p*=0.021) and PCD (*p* < 0.001). McGrath also had the fewest number of tooth compressions in comparison with MAC (*p*=0.006) and PCD (*p* < 0.001).

The ease of use averaged 27 (IQR: 14–38) for MAC, 20 (IQR: 11–24) for McGrath, and 39 (IQR: 24–49) for PCD.

### 3.4. Scenario D: Cervical Collar Stabilization and Tongue Oedema

Detailed results obtained in scenario D are presented in Table 4. The success rate with MAC was 32%, compared to 69% for McGrath and 58% for PCD.

The intubation time was 26 (IQR: 20–29) seconds for McGrath, compared to 54 (IQR: 39–71) seconds for MAC and 45 (IQR: 33–56) seconds for PCD.

The Cormack-Lehane score was the best for McGrath. The highest POGO score was obtained for McGrath and equaled 86%, compared to 80% for PCD and 35% for MAC.

Endotracheal intubation with McGrath was associated with the fewest optimization manoeuvres in comparison with MAC (*p* < 0.001) and PCD (*p*=0.023). McGrath also had the fewest number of tooth compressions as compared with MAC (*p* < 0.001) and PCD (*p* < 0.001).

The ease of use averaged 81 (IQR: 63–90) for MAC, 34 (IQR: 28–48) for McGrath, and 57 (IQR: 45–75) for PCD.

### 3.5. Scenario E: Cervical Collar Stabilization and Pharyngeal Obstruction

Detailed results of the parameters obtained in scenario E are presented in Table 5. The success rate was 32% with MAC, compared to 64% with McGrath and 62% with PCD.

The intubation time with the studied methods was the least for McGrath and equaled 19 (IQR: 18–26) seconds; the values were 28 (IQR: 25–39) seconds for MAC and 34 (IQR: 27–45) seconds for PCD.

Again, the best Cormack-Lehane score and highest POGO score were obtained for McGrath.

Endotracheal intubation with McGrath was associated with the fewest optimization manoeuvres in comparison with MAC (*p* < 0.001) and PCD (*p*=0.012). McGrath also had the fewest number of tooth compressions as compared with MAC (*p* < 0.001) and PCD (*p* < 0.001).

The ease of use averaged 68 (IQR: 51–80) for MAC, 31 (IQR: 28–45) for McGrath, and 51 (IQR: 36–64) for PCD.

## 4. Discussion

The most important finding of our randomized cross-over simulation study is the superiority of the McGrath video laryngoscope compared to conventional Macintosh laryngoscope intubation, considering the overall success rate, duration of intubation attempt, Cormack-Lehane grade, POGO score, number of optimization manoeuvres, severity of dental compression, and ease of use in all 5 tested scenarios of normal and difficult airways.

Overall, the success rate for MAC ranged between 15% in scenario E and 99% in scenario A. While the success rate in the normal airway is acceptable, a success rate of only 15% is far from acceptable. Consequently, MAC should not serve as the first airway intubation device for paramedics.

For PCD, the success rate varied between 57 and 97% in the several airway scenarios. It is worth noting that, in all difficult airway scenarios, the overall success rate for McGrath was between 69% and 94%. We noticed that paramedics performed endotracheal intubation with best results when using McGrath as compared with PCD and MAC. In contrast to our results, Altun et al., in a simulation study on Macintosh, McGrath, McCoy, and C-MAC laryngoscopes among 41 anaesthesiology residents, revealed that McGrath offered the longest intubation time, especially in a difficult airway (tongue oedema) scenario [11]. In an observational study in a prehospital setting, the McGrath MAC video laryngoscope first-pass success rate did not change when compared with the previous period of using Macintosh laryngoscope; however, the post–rapid sequence induction first-pass success rate was significantly higher. The authors concluded that gastric content, blood, or secretion in the airway resulted in reduced vision when using the McGrath MAC video laryngoscope [13].

Choi et al. in a manikin study compared tracheal intubation by novice users applying the McGrath series 5 video laryngoscope versus the Macintosh laryngoscope in a cervical immobilized manikin and revealed that the first-attempt success rate was higher for the McGrath compared with the Macintosh laryngoscope in cervical immobilizations (84% versus 48%, resp.; *p*=0.019). In our study in scenario D: cervical collar stabilization and tongue oedema, the values of 69% versus 32% (*p* < 0.001) were obtained for McGrath and Macintosh laryngoscope [14].

The duration of the intubation attempt is a clinically important factor and differences of 15 seconds or more can be clinically important, especially in severe hypoxia or cardiac arrest patients. In our study, the shortest time of intubation attempt among all difficult airway scenarios was obtained for McGrath intubation; however, only in normal airway the time was not statistically significantly shorter (17 seconds versus 18 seconds for MAC and McGrath; *p*=0.899). In the presented study, in some difficult airway scenarios, the time of intubation attempt was shorter as compared with MAC (scenarios B and D). This finding is generally supported by other studies. Bag et al. found the mean time necessary for intubation equaled 21.10 ± 5.64 seconds for Truview and 15.79 ± 2.76 seconds for Macintosh [11]. A clinical study conducted by Bhola et al. comparing McGrath and Truview PCD reported that the time to successful intubation was shorter with the McGrath video laryngoscope when compared with Truview (30.02 seconds versus 38.72 seconds) but there was no significant difference between the laryngoscopic views obtained in both groups [15]. Singh et al. published the results of their study on intubation using Truview PCD, C-MAC, and Macintosh laryngoscopes in paediatric patients. They concluded that the intubation time was 19.2 seconds for Truview PCD and 10.7 s for Macintosh [16].

From a clinical point of view, each intubation attempt is important, with repeated intubation attempts leading to an increased risk of complications including bleeding and oedema of the airways, which reduce the chance of successful endotracheal intubation [17]. Alvis et al. compared the performance of the McGrath MAC and King Vision laryngoscope systems for endotracheal intubation in adult patients with predicted normal airways when used by experienced laryngoscopists with a limited prior video laryngoscopy experience. The first-attempt success rate was higher in the McGrath MAC group than in the King Vision group (100% versus 89%; *p* < 0.01), and the times to endotracheal intubation were significantly shorter for McGrath [18].

The Cormack-Lehane grade in our study was assessed by the paramedics. In all difficult airway scenarios, better glottis view was obtained for McGrath compared with PCD and MAC. This finding is supported by other studies, including a meta-analysis by Hoshijima et al., who revealed that McGrath was superior to Macintosh in terms of glottic visualization [19].

In our study, POGO score was better for McGrath compared with MAC and PCD. This finding is concordant with other studies. Singh et al. published the results of their study on intubation using Truview PCD, C-MAC, and Macintosh laryngoscopes in paediatric patients. They concluded that POGO scores were significantly better with Truview PCD than with Macintosh laryngoscopes (94.7 ± 12.9 versus 85.1 ± 17.1; *p* < 0.01). The number of necessary external manoeuvres was fewer in the Truview PCD than in the Macintosh group [16].

As a limitation, higher success rates and shorter intubation times obtained with MAC compared with video laryngoscopes may be due to greater experience with MAC. Expertise with standard direct laryngoscopy does not translate to that with video laryngoscopy, and separate training and experience with video laryngoscopy are required. Our study included only paramedics experienced with MAC, with no prior experience in any video laryngoscopes. We enrolled paramedics because this group of medical personnel must provide airway management including endotracheal intubation in out-of-hospital settings with no support from experienced personnel, for example, anaesthesiologists.

A further limitation is the nature of any manikin study. Results of any manikin study need to be confirmed in clinical studies, which is extremely challenging due to ethical concerns, especially if investigated by less experienced providers like paramedics. However, manikin studies are generally considered reliable. Another limitation is the potential of bias, as it is impossible to blind participating paramedics. The POGO score and ease of use were subjective measures. The application of manikins allows for enough statistical power while performing cross-over studies with no risk for humans.

In conclusion, in this randomized cross-over simulation study performed among a group of paramedics, the McGrath video laryngoscope was demonstrated to be superior to the Truview PCD video laryngoscope and direct laryngoscopy guided intubation using a Macintosh laryngoscope in terms of success rate, duration of intubation attempt, Cormack-Lehane grade, POGO score, number of optimization manoeuvres, severity of dental compression, and ease of use.

## Figures and Tables

**Table 1:** Intubation details in scenario A normal airway. Data are presented as median (IQR) or as number (%).

Parameter	A	B	C	*p* values for between-device differences			*p*
	Macintosh	McGrath	TruView PCD	A versus B	A versus C	B versus C	
*Success rate of intubation attempt (%)*	92 (99%)	93 (100%)	87 (94%)	0.127	0.644	0.761	0.011

*Duration of 1st intubation attempt (sec)*	17 (16–21)	18 (16–21)	27 (23–34)	1.0	**<0.001**	**<0.001**	**<0.001**

*Cormack-Lehane grade*	87 (94%)	93 (100%)	93	1.0	1.0	1.0	1.0
1	6 (7%)	0 (0%)	(100%)				
2	0 (0%)	0 (0%)	0 (0%)				
3	0 (0%)	0 (0%)	0 (0%)				
4	1 (1–1)	1 (1–1)	0 (0%)				
Median (IQR)			1 (1–1)				

*POGO score*	81 (78–100)	100 (95–100)	100 (90–100)	**0.239**	**0.030**	1.0	**0.028**

*Number of optimization manoeuvres (%)*	84 (90%)	92 (99%)	76	1.0	1.0	0.561	0.982
0	9 (10%)	1 (1 %)	(81%)				
1	0 (0%)	0 (0%)	16 (19%)				
2	0 (0–0)	0 (0–0)	1 (1%)				
Median (IQR)			0 (0–0)				

*Severity of dental compression (%)*	36 (39%)	48 (52%)	2 (2%)	0.770	**<0.001**	**<0.001**	**<0.001**
0	57 (61%)	45 (48%)	20				
1	0 (0%)	0 (0%)	(22%)				
2	1 (0–1)	0 (0–1)	71				
Median (IQR)			(76%) 2 (2–2)				

*Ease of use (1–100)*	24 (10–27)	20 (11–23)	31 (17–35)	0.671	**<0.001**	**<0.001**	**<0.001**

**Table 2:** Intubation details in scenario B tongue oedema. Data are presented as median (IQR) or as number (%).

Parameter	A	B	C	*p* values for between-device differences			*p*
	Macintosh	McGrath	TruView PCD	A versus B	A versus C	B versus C	
*Success rate of intubation attempt (%)*	57 (61%)	90 (97%)	90 (97%)	**<0.001**	**<0.001**	1.0	**<0.001**

*Duration of 1st intubation attempt (s)*	44 (24–46)	22 (20–27)	39 (30–57)	0.822	0.392	0.138	0.126

*Cormack-Lehane grade*	0 (0%)	77 (83%)	70 (75%)	**<0.001**	**<0.001**	1.0	**<0.001**
1	3 (3%)	16 (17%)	23 (25%)				
2	86 (93%)	0 (0%)	0 (0%)				
3	4 (4%)	0 (0%)	0 (0%)				
4	3 (3–3)	1 (1–1)	1 (1–1)				
Median (IQR)							

*POGO score*	37 (29–49)	93 (80–100)	90 (82–95)	**<0.001**	**<0.001**	0.117	**<0.001**

*Number of optimization manoeuvres (%)*	0 (0%)	19 (20%)	0 (0%)	**<0.001**	1.0	**<0.001**	**<0.001**
0	17 (19%)	29 (31%)	14 (18%)				
1	76 (81%)	45 (48%)	79 (82%)				
2	2 (2–2)	1 (1–2)	2 (2–2)				
Median (IQR)							

*Severity of dental compression (%)*				**<0.001**	1.0	**<0.001**	**<0.001**
0	0 (0%)	40 (43%)	0 (0%)				
1	12 (13%)	44 (47%)	20 (22%)				
2	81 (87%)	9 (10%)	73 (79%)				
Median (IQR)	2 (2–2)	1 (0–1)	2 (2–2)				

*Ease of use (1–100)*	74 (50–80)	34 (26–47)	46 (38–61)	**<0.001**	**<0.001**	**<0.001**	**<0.001**

**Table 3:** Intubation details in scenario C pharyngeal obstruction. Data are presented as median (IQR) or as number (%).

Parameter	A	B	C	*p* values for between-device differences			*p*
	Macintosh	McGrath	TruView PCD	A versus B	A versus C	B versus C	
*Success rate of intubation attempt (%)*	69 (74%)	90 (97%)	67 (72%)	**0.023**	1.0	**0.011**	**<0.001**

*Duration of 1st intubation attempt (s)*	21 (19–29)	18 (18–25)	30 (23–39)	**0.003**	**0.001**	**<0.001**	**<0.001**

*Cormack-Lehane grade*	82 (88%)	93 (100%)	92 (99%)	1.0	1.0	1.0	1.0
1	11 (12%)	0 (0%)	1 (1%)				
2	0 (0%)	0 (0%)	0 (0%)				
3	0 (0%)	0 (0%)	0 (0%)				
4	1 (1–1)	1 (1–1)	1 (1–1)				
Median (IQR)							

*POGO score*	80 (78–100)	100 (95–100)	100 (84–100)	**<0.001**	**<0.001**	0.817	**<0.001**

*Number of optimization manoeuvres (%)*	73 (79%)	92 (99%)	39 (42%)	**0.021**	**<0.001**	**<0.001**	**<0.001**
0	6 (7%)	1 (1%)	46 (50%)				
1	14 (15.0%)	0 (0%)	8 (9%)				
2	0 (0–0)	0 (0–0)	1 (0–1)				
Median (IQR)							

*Severity of dental compression (%)*	21 (23%)	37 (40%)	2 (2%)	**0.006**	**<0.001**	**<0.001**	**<0.001**
0	53 (57%)	53 (57%)	41 (44%)				
1	19 (21%)	3 (3%)	50 (54%)				
2	1 (1–1)	1 (0–1)	2 (1–2)				
Median (IQR)							

*Ease of use (1–100)*	27 (14–38)	20 (11–24)	39 (24–49)	**<0.001**	**<0.001**	**<0.001**	**<0.001**

**Table 4:** Intubation details in scenario D cervical collar stabilization and tongue oedema. Data are presented as median (IQR) or as number (%).

Parameter	A	B	C	*p* values for between-device differences			*p*
	Macintosh	McGrath	TruView PCD	A versus B	A versus C	B versus C	
*Success rate of intubation attempt (%)*	30 (32%)	64 (69%)	58 (62%)	**<0.001**	**<0.001**	1.0	**<0.001**

*Duration of 1st intubation attempt (s)*	54 (39–71)	26 (20–29)	45 (33–56)	**<0.001**	**<0.001**	**<0.001**	**<0.001**

*Cormack-Lehane grade*	0 (0%)	64 (69%)	50 (54%)	**<0.001**	**<0.001**	0.798	**<0.001**
1	3 (3%)	29 (31%)	43 (46%)				
2	71 (76%)	0 (0%)	0 (0%)				
3	19 (21%)	0 (0%)	0 (0%)				
4	3 (3–3)	1 (1–2)	1 (1–2)				
Median (IQR)							

*POGO score*	35 (29–42)	86 (75–94)	80 (75–91)	**<0.001**	**<0.001**	0.725	**<0.001**

*Number of optimization manoeuvres (%)*	4 (4%)	11 (12%)	1 (1%)	**<0.001**	**0.009**	**0.023**	**<0.001**
0	10 (20%)	47 (51%)	39 (42%)				
1	79 (85%)	35 (38%)	533 (57%)				
2	2 (2–2)	1 (1–2)	2 (1–2)				
Median (IQR)							

*Severity of dental compression (%)*	0 (0%)	0 (0%)	0 (0%)	**<0.001**	0.489	**<0.001**	**<0.001**
0	2 (2%)	76 (82%)	13 (14%)				
1	91 (98%)	17 (18%)	80 (86%)				
2	2 (2–2)	1 (1–1)	2 (2–2)				
Median (IQR)							

*Ease of use (1–100)*	81 (63–90)	34 (28–48)	57 (45–75)	**<0.001**	**<0.001**	**<0.001**	**<0.001**

**Table 5:** Intubation details in scenario E cervical collar stabilization and pharyngeal obstruction. Data are presented as median (IQR) or as number (%).

Parameter	A	B	C	*p* values for between-device differences			*p*
	Macintosh	McGrath	TruView PCD	A versus B	A versus C	B versus C	
*Success rate of intubation attempt (%)*	14 (15%)	87 (94%)	53 (57%)	**<0.001**	**<0.001**	**<0.001**	**<0.001**

*Duration of 1st intubation attempt (s)*	28 (25–39)	19 (18–26)	34 (27–45)	**0.059**	0.787	**0.009**	**0.018**

*Cormack-Lehane grade*	0 (0%)	79 (84%)	40 (43%)	**<0.001**	**<0.001**	**0.031**	**<0.001**
1	31 (33%)	14 (16%)	53 (57%)				
2	58 (62%)	0 (0%)	0 (0%)				
3	4 (4%)	0 (0%)	0 (0%)				
4	3 (2–3)	1 (1–1)	1 (1–2)				
Median (IQR)							

*POGO score*	45 (29–63)	98 (90–100)	92 (83–98)	**<0.001**	**<0.001**	**0.003**	**<0.001**

*Number of optimization manoeuvres (%)*	10 (11%)	31 (33%)	19 (20%)	**<0.001**	**0.005**	**0.012**	**<0.001**
0	21 (23%)	45 (48%)	37 (40%)				
1	62 (67%)	17 (18%)	37 (40%)				
2	2 (1–2)	1 (0–1)	1 (1–2)				
Median (IQR)							

*Severity of dental compression (%)*	0 (0%)	0 (0%)	0 (0%)	**<0.001**	**0.007**	**<0.001**	**<0.001**
0	7 (8%)	90 (97%)	31 (33%)				
1	86 (93%)	3 (3%)	62 (67%)				
2	2 (2–2)	1 (1–1)	2 (1–2)				
Median (IQR)							

*Ease of use (1–100)*	68 (51–80)	31 (28–45)	51 (36–64)	**<0.001**	**<0.001**	**<0.001**	**<0.001**

## Data Availability

The data used to support the findings of this study are available from the corresponding author upon request.

## Supplementary Materials

Supplementary Materials Supplementary figure 1: median POGO score.

## References

[1] Ruetzler K., Roessler B., Potura L. (2011). Performance and skill retention of intubation by paramedics using seven different airway devices-A manikin study. *Resuscitation*.

[2] Goliasch G., Ruetzler A., Fischer H., Frass M., Sessler D. I., Ruetzler K. (2013). Evaluation of advanced airway management in absolutely inexperienced hands. *European Journal of Emergency Medicine*.

[3] Savino P. B., Reichelderfer S., Mercer M. P., Wang R. C., Sporer K. A. (2017). Direct versus video laryngoscopy for prehospital intubation: a systematic review and meta-analysis. *Academic Emergency Medicine*.

[4] Piegeler T., Roessler B., Goliasch G. (2016). Evaluation of six different airway devices regarding regurgitation and pulmonary aspiration during cardio-pulmonary resuscitation (CPR)- a human cadaver pilot study. *Resuscitation*.

[5] Ruetzler K., Guzzella S. E., Tscholl D. W. (2017). Blind intubation through self-pressurized, disposable supraglottic airway laryngeal intubation masks. *Anesthesiology*.

[6] Ruetzler K., Imach S., Weiss M., Haas T., Schmidt A. R. (2015). Vergleich von fünf Videolaryngoskopen und direkter konventioneller Laryngoskopie. *Der Anaesthesist*.

[7] Zhu H., Liu J., Suo L., Zhou C., Sun Y., Jiang H. (2019). A randomized controlled comparison of non-channeled king vision, McGrath MAC video laryngoscope and Macintosh direct laryngoscope for nasotracheal intubation in patients with predicted difficult intubations. *BMC Anesthesiology*.

[8] Szarpak L., Karczewska K., Evrin T., Kurowski A., Czyzewski L. (2015). Comparison of intubation through the McGrath MAC, GlideScope, AirTraq, and Miller Laryngoscope by paramedics during child CPR: a randomized crossover manikin trial. *The American Journal of Emergency Medicine*.

[9] Goyagi T., Nishikawa T. (2013). Comparative performance of Airway Scope, Trueview EVO2, and Fibertech video laryngoscope used by inexperienced medical students in a simulated manikin with normal and difficult airways. *Masui*.

[10] Smereka J., Czyzewski L., Szarpak L., Ladny J. R. (2017). Comparison between the TrueView EVO2 PCD and direct laryngoscopy for endotracheal intubation performed by paramedics: preliminary data. *The American Journal of Emergency Medicine*.

[11] Altun D., Ozkan‐Seyhan T., Orhan‐Sungur M., Sivrikoz N., Camci E. (2016). Comparison of 4 laryngoscopes in 2 difficult airway scenarios: a randomized crossover simulation-based study. *Simulation in Healthcare: The Journal of the Society for Simulation in Healthcare*.

[12] Biro P., Ruetzler K. (2015). The reflective intubation manoeuvre increases success rate in moderately difficult direct laryngoscopy. *European Journal of Anaesthesiology*.

[13] Rhode M. G., Vandborg M. P., Bladt V., Rognas L. (2016). Video laryngoscopy in pre-hospital critical care - a quality improvement study. *Scandinavian Journal of Trauma, Resuscitation and Emergency Medicine*.

[14] Choi J. W., Kim J. A., Jung H. J., Kim W. H. (2016). Tracheal intubation with a McGrath series 5 video laryngoscope by novice personnel in a cervical-immobilized manikin. *The Journal of Emergency Medicine*.

[15] Bhola R., Bhalla S., Gupta R., Singh I., Kumar S. (2014). Tracheal intubation in patients with cervical spine immobilization: a comparison of McGrathvideo laryngoscope and Truview EVO2laryngoscope. *Indian Journal of Anaesthesia*.

[16] Singh R., Kumar N., Jain A. (2017). A randomised trial to compare Truview PCD, C-MAC and Macintosh laryngoscopes in paediatric airway management. *Asian Journal of Anesthesiology*.

[17] Hypes C., Sakles J., Joshi R. (2017). Failure to achieve first attempt success at intubation using video laryngoscopy is associated with increased complications. *Internal and Emergency Medicine*.

[18] Alvis B. D., Hester D., Watson D., Higgins M., St Jacques P. (2016). Randomized controlled trial comparing the McGrath MAC video laryngoscope with the King Vision video laryngoscope in adult patients. *Minerva anestesiologica*.

[19] Hoshijima H., Mihara T., Maruyama K. (2018). McGrath videolaryngoscope versus Macintosh laryngoscope for tracheal intubation: a systematic review and meta-analysis with trial sequential analysis. *Journal of Clinical Anesthesia*.

