# Supplementary figures and images for: Comparison of Direct and Video Laryngoscopes during Different Airway Scenarios Performed by Experienced Paramedics: A Randomized Cross-Over Manikin Study
Source: Biomed Res Int. 2020 Feb 18;2020:5382739. doi: 10.1155/2020/5382739 (PMC7049447; doi:10.1155/2020/5382739)

**Supplementary figure 1.** Median POGO score.

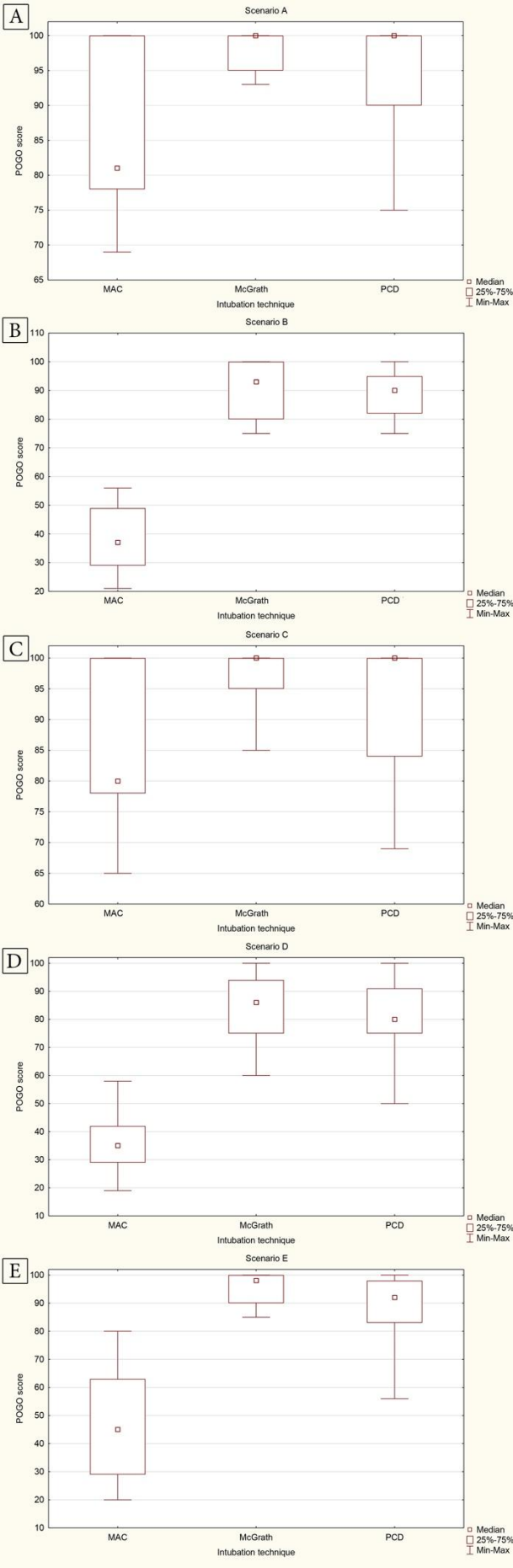

Supplement: Supplementary Materials — Supplementary figure 1: median POGO score. [file 5382739.f1.pdf]
